# Structured Exercise Interventions and Hepatic–Metabolic Outcomes in Adults with MASLD: A Narrative Review of Randomized Controlled Trials

**DOI:** 10.3390/ijms27072941

**Published:** 2026-03-24

**Authors:** Tuva Marie Lindstad, Shirin Pourteymour, Sindre Lee-Ødegård, Christian André Drevon, Frode Amador Norheim

**Affiliations:** 1 Department of Nutrition, Institute of Basic Medical Sciences, Faculty of Medicine, University of Oslo, P.O. Box 0317 Oslo, Norway; t.m.lindstad@studmed.uio.no (T.M.L.); shirin.pourteymour@medisin.uio.no (S.P.); c.a.drevon@medisin.uio.no (C.A.D.); 2 Institute of Clinical Medicine, Faculty of Medicine, University of Oslo, P.O. Box 0318 Oslo, Norway; sindre.lee-odegard@medisin.uio.no; 3 Department of Endocrinology, Obesity and Preventive Medicine, Oslo University Hospital, P.O. Box 0424 Oslo, Norway; 4 Vitas Ltd., Oslo Science Park, P.O. Box 0349 Oslo, Norway

**Keywords:** MASLD, fatty liver, exercise, aerobic training, resistance training, physical activity, hepatic steatosis

## Abstract

Metabolic dysfunction-associated steatotic liver disease (MASLD, introduced in 2023), formerly termed non-alcoholic fatty liver disease (NAFLD), is a leading cause of chronic liver disease worldwide and is closely linked to obesity, insulin resistance, and cardiometabolic dysfunction. Exercise is widely recommended as a cornerstone of MASLD management; however, the magnitude of its hepatic and metabolic benefits and the underlying molecular mechanisms remain incompletely defined. We aim to synthesize evidence from randomized controlled trials assessing how structured exercise interventions influence hepatic steatosis and metabolic dysfunction in adults with MASLD. A targeted search of PubMed from database inception to February 2025 identified eligible trials, of which eleven were included in the qualitative synthesis. Across studies, aerobic and resistance exercise interventions were consistently associated with reductions in hepatic fat content, improvements in plasma lipid profiles and liver enzyme concentrations, and enhanced indices of insulin sensitivity, frequently occurring independently of substantial weight loss. Mechanistically, exercise-induced activation of pathways related to mitochondrial function, lipid oxidation, inflammation modulation, and insulin signaling likely contributes to these benefits. Despite heterogeneity in intervention design, duration, and outcome assessment, the collective evidence supports structured exercise as an effective non-pharmacological strategy for improving hepatic steatosis and metabolic dysfunction in MASLD. Future studies integrating molecular biomarkers with clinical endpoints are warranted to refine exercise prescriptions and elucidate mechanisms of therapeutic response.

## 1. Introduction

Metabolic dysfunction-associated fatty liver disease (MAFLD) is the most prevalent form of steatotic liver disease (SLD) and is characterized by excess hepatic fat accumulation along with metabolic dysfunction. The pooled global prevalence of MAFLD was 29.8% as revealed in a systematic review and meta-analysis including 5.4 mill individuals. The prevalence increased 15.4% from 1991 to 2019, when Europe exhibited 1.1% increase per year [1].

Metabolic dysfunction-associated steatotic liver disease (MASLD) is defined by the presence of hepatic steatosis together with at least one of the five designated cardiometabolic risk factors outlined in the 2023 consensus. Hepatic steatosis is typically quantified using non-invasive imaging modalities, with magnetic resonance imaging (MRI) considered the gold standard for assessing liver fat content [2,3]. MRI is the gold standard for non-invasive quantification of hepatic steatosis and is often used to assess liver fat content.

Since the initial description of fatty liver disease, the condition was referred to as non-alcoholic fatty liver disease (NAFLD) due to its resemblance to alcohol-related liver injury. In 2020, the term was revised to metabolic dysfunction-associated fatty liver disease (MAFLD) to emphasize the central role of metabolic dysfunction in disease development [2,3]. In 2023, an international consensus statement has introduced the term (MASLD) as the preferred nomenclature, aligning the definition with cardiometabolic risk factors and simplifying diagnostic classification. We acknowledge the historical use of NAFLD and MAFLD but adopt MASLD as the primary terminology for clarity and consistency.

MASLD is linked to sarcopenia and metabolic risk factors, many of which are driven by unhealthy diet and physical inactivity. Sarcopenia, characterized by loss of muscle mass and strength, is an independent risk factor for both the development of MASLD and its progression to metabolic dysfunction-associated steatohepatitis (MASH) [4,5]. Sarcopenia and MASLD often share common traits, including altered body composition and increased visceral adipose tissue (VAT).

VAT is an independent predictor of liver damage, with a well-established role in hepatic inflammation and fibrosis [6]. Increased VAT contributes to the disease by releasing free fatty acids (FFAs) promoting hepatic fat accumulation and inflammation [7]. Moreover, the continuous release of pro-inflammatory molecules and FFAs from VAT can lead to insulin resistance, oxidative stress, and progression to steatohepatitis. However, recent data suggests that accumulation of hepatic fat may also occur independently of VAT expansion [6].

The fact that MASLD is a multifactorial disease has made it challenging to develop pharmacological options. Its progression to MASH can be affected by mitochondrial dysfunction, dysregulated lipogenesis, unresolved endoplasmic reticulum stress, and impaired autophagy [8,9]. Additional work has shown that metabolic signaling pathways, including BDNF, mTOR, ketone metabolism, and thyroid hormone regulation—interact and may influence MASLD pathophysiology [10]. Currently, pharmacologic options for MASLD remain limited; resmetirom, a selective thyroid hormone receptor-β agonist, is the first agent to receive FDA approval for the treatment of MASH with moderate to advanced fibrosis [11,12], whereas other candidates like semaglutide (GLP-1 receptor agonist) and lanifibranor (pan-PPAR agonist) are in late-phase clinical trials [13,14]. Lifestyle interventions are recommended as the cornerstone of both prevention and treatment [15,16,17]. For overweight or obese subjects with MASLD, achieving a weight loss of 5–10% is considered essential for improving liver health [16], with dietary modification being the most common approach. To reduce steatosis significantly, a minimum weight loss of 3–5% is required [15]. Non-obese individuals with MASLD may also benefit from weight loss, although they require less weight reduction than obese individuals [18].

Both aerobic and resistance training can effectively improve hepatic steatosis in patients with MASLD [19,20]. Aerobic exercise includes activities that improve cardiorespiratory fitness, whereas resistance training increases strength and mass of skeletal muscles. Although weight loss due to an energy-restrictive diet is effective, it may not fully capture the extensive metabolic benefits provided by exercise, particularly concerning insulin sensitivity. Exercise does not only increase energy expenditure but also promotes favorable changes in body composition by increasing lean muscle mass and reducing VAT, both of which are crucial for managing different chronic diseases [21]. Furthermore, a recent study reported that aerobic exercise had considerable weight-loss-independent benefits on both hepatic steatosis and hepatic stiffness [22]. Underlying mechanisms through which different exercise modalities influence MASLD are likely to differ [23]. Although numerous studies have examined lifestyle interventions that include exercise, the optimal dose, intensity, and type of training remain uncertain [24].

Despite consistent evidence that exercise improves hepatic steatosis and metabolic dysfunction, substantial variability in study design, training protocols, and outcome measures limits direct comparison across trials. To address these inconsistencies, we synthesize findings from randomized controlled trials to evaluate how different exercise modalities affect hepatic steatosis and key metabolic outcomes in adults with MASLD.

## 2. Results

### 2.1. Study Characteristics

A total of 11 randomized controlled trials met the inclusion criteria and were included in the qualitative synthesis (Table S1). The characteristics of the included studies are summarized in Supplementary Table S1. Sample sizes ranged from 18 to 220 participants. Most trials enrolled both men and women, with one study including male participants only. Participants were 19 years of age or older, with a mean age of 51.2 years, and the average body mass index ranged from 27.8 to 40.0 kg/m^2^. All participants were diagnosed with primary MASLD, either alone or in combination with comorbidities such as type 2 diabetes mellitus or obesity. One study additionally included a control group without MASLD [25]. Given the heterogeneity of study populations, exercise modalities, and outcome measures, findings should be interpreted with caution.

### 2.2. Intervention Duration and Outcomes

The duration of exercise interventions ranged from two to 12 months, with sessions conducted weekly or up to five times per week. Each session typically lasted no more than one hour. In three studies, participants also received dietary recommendations or followed a prescribed diet along with the exercise intervention. The primary outcomes included intrahepatic triglycerides, plasma liver enzymes, and lipid profiles. Most RCTs reported reductions in liver fat, although the magnitude and statistical significance varied across studies. Even in trials where no significant differences were observed between intervention and control groups, improvements were still noted within the exercise groups when comparing pre- and post-intervention values. Several studies showed within-group reductions in hepatic fat that did not translate into significant between-group differences, suggesting that some improvement may reflect non-specific or within-group effects rather than clear superiority over control conditions (Table 1).

### 2.3. Intervention Types and Comparisons

Most trials compared different types or intensities of physical exercise either alone or in combination with a specific diet. The exercise interventions included high-intensity intervals, resistance training, and vigorous or moderate aerobic training, tested either against other intervention groups or a control group. The study by Bacchi et al. [26] focused solely on a direct comparison between aerobic and resistance training. Franco et al. [27] evaluated the effects of the traditional Mediterranean diet with or without different exercise programs. In this trial, PA1 refers to an aerobic training program, and PA2 refers to a combined aerobic and resistance training program.

### 2.4. Effects of Aerobic Training

Nine of the included RCTs assessed the effects of aerobic exercise, with most reporting reductions in intrahepatic fat content [25,26,27,28,29,30,31,32,33]. However, not all studies demonstrated significant between-group differences compared with control conditions, and several findings reflected within-group improvements only. Of these trials, five (55.6%) also reported weight loss, with three incorporating dietary changes. Sullivan et al. [33] observed a significant reduction in intrahepatic triglycerides (−10.3%, *p* < 0.05) after a four-month intervention, even in the absence of weight loss or dietary modifications, compared to controls. Abdelbasset et al. [29] reported significant reductions in intrahepatic triglycerides within the exercise groups—high-intensity interval training (−2.3%, *p* < 0.05) and moderate-intensity aerobic training (−2.4%, *p* < 0.05), although these changes did not translate into significant between-group differences. These interventions were also associated with improved insulin sensitivity, plasma lipid profiles, and liver enzymes within the exercise groups [29].

Cuthbertson et al. [30], Hallsworth et al. [31] and Shojaee-Moradie et al. [32] reported significantly reduced liver fat in the aerobic groups as compared to controls (*p* < 0.05). Specifically, Cuthbertson et al. [30] and Shojaee-Moradie et al. [32] found reductions of 9.3% and 10.7%, respectively, after four months of exercise interventions. Hallsworth et al. [31] observed a 2.8% reduction following a three-month intervention. In contrast, Pugh et al. [25] found only a non-significant trend toward reduced intrahepatic fat (8.4%, *p* > 0.05) after a four-month intervention, with no significant change in other MAFLD-related parameters. Overall, aerobic exercise interventions showed beneficial effects on hepatic fat, although the magnitude and statistical significance varied across studies. Improvements observed only within exercise groups should be interpreted cautiously, as they may reflect non-specific effects rather than clear superiority over control conditions.

**Table 1:** Findings on the effects of exercise in MAFLD patients after interventions.

Reference	Participants	Interventions	Weight Loss/Change	Insulin Resistance	Lipid Profile	Liver Enzymes	Liver Fat	Conclusion
Shojaee-Moradie et al. [32]	Sedentary, MAFLD	Aerobic training vs. control	−3.95% ***	NA	NS change, LDL improved within groups	NS change, improved within group	−10.7% * IHCL	Aerobic exercise reduced intrahepatic fat, and liver enzymes and insulin resistance improved within the intervention group.
Zelber-Sagi et al. [34]	MAFLD	Resistance training vs. stretching	−0.39 kg vs. 0.33 kg *	NS change	−8.61 mg/dL cholesterol *	NS change	−0.25 * HRI score	Resistance training improved hepatic fat compared to stretching. HRI > 1.5 indicates fatty liver. The HRI score was 1.86 in RT group
Abdelbasset et al. [29]	Obese, diabetic, MAFLD	HIIT vs. aerobic training	NA	NS change	NS change	NS change	NS change	After the intervention both exercise groups reduced IHTG and improved lipid profile.
		HIIT (within group)	NA	−0.8 * HOMA-IR	Reduction in all lipids *	−4.1 * IU/L ALT	−2.3% * IHTG	
		Aerobic training (within group)	NA	−0.8 * HOMA-IR	Reduction all lipids *	−3.7 * IU/L ALT	−2.4% * IHTG	
Cuthbertson et al. [30]	MAFLD	Aerobic training vs. control	−2.5% *	−0.43 vs. 0.03 * HOMA2-IR	NS change	NS change	−9.3% * liver fat (% CH_2_/H_2_O)	Aerobic exercise improved intrahepatic fat.
Bacchi et al. [26]	Sedentary, T2DM, MAFLD	Resistance training vs. aerobic training	NA	NA	NS change, improved within group	NS change	NS change, improved within group	Both groups reduced intrahepatic fat (equally effective), and hepatic steatosis disappeared in ¼ of the patients.
		Resistance training (within group)	NA	NA	−21.0 mg/dL * TG	NS change	−25.8% *** liver fat	
		Aerobic training (within group)	NA	NA	−13.7 mg/dL * TG	NS change	−32.8% *** liver fat	
Franco et al. [27]	MAFLD	LGIMD vs. control	NA	NS change	NS change	NA	−55.81 CAP value (NS change)	All interventions arms, except LGIMD showed statistic significantly decrease in NAFLD score. PA1 and PA1 + LGIMD had the greatest effect. PA1 + LGIMD showed the greatest effect over time. Intervention had a better effect in the severe NAFLD group
		PA1 vs. control	NA	NS change	NS change	NA	−166.35 * CAP value	
		PA2 vs. control	NA	NS change	NS change	NA	−78.02 * CAP	
		PA1 + LGIMD vs. control	NA	NS change	NS change	NA	−94.10 * CAP	
		PA2 + LGIMD vs. control	NA	NS change	NS change	NA	−76.37 * CAP	
Zhang et al. [28]	MAFLD, China	Vigorous-moderate aerobic training vs. moderate aerobic training	NA	NA	NS change	1.8 U/L ** AST (6-mo)	−0.8% IHTG (6-mo) −0.4% IHTG (12-mo) NS change	Both exercise groups reduced IHTG, and ALT improved with vigorous exercise compared to moderate exercise during 6-mo. No significant difference was found between exercise groups.
		Vigorous-moderate vs. control	−4.33 kg *** (6-mo) −3.19 kg *** (12-mo)	NA	NS change	NS change	−5.0% *** IHTG (6-mo) −3.8% *** IHTG (12-mo)	
		Moderate vs. control	−2.61 kg *** (12-mo)	NA	NS change	NS change	−4.2% *** IHTG (6-mo) −3.5% *** IHTG (12-mo)	
Hallsworth et al. [31].	MAFLD	HIIT vs. control	−1.4 kg *	NS change	NS change	−10 U/L vs. 4 U/L ALT * −3 U/L vs. 4 U/L AST *	−2.8%* IHL	HIIT reduced intrahepatic lipids and plasma ALT and AST.
Sullivan et al. [33]	MAFLD	Aerobic training vs. control	NS change	NA	NS change	−6.3 U/L vs. 5.6 U/L ALT *	−10.3% * IHTG	Aerobic exercise without weight loss improved both ALT and intrahepatic triglyceride content.
Pugh et al. [25]	Obese, MAFLD	Aerobic training vs. control	−2.1 kg (NS change)	NS change	NS change	NS change	−8.4% liver fat (%CH_2_/H_2_O) NS change	Aerobic exercise improved flow-mediated dilation, but no significant difference in liver fat.

NA = not assessed; CAP = controlled attenuation parameter; HRI = hepatorenal-ultrasound index; IHTG = intrahepatic triglycerides; IHCL = intrahepatocellular lipids; IHL = intrahepatic lipid; CH_2_/H_2_O = proton density fat fraction ratio; NS = non-significant; PA1 = aerobic training; PA2 = aerobic and resistance training; LGIMD = low-glycemic index Mediterranean diet. * *p* < 0.05, ** *p* < 0.005, *** *p* < 0.001.

### 2.5. Effects of Resistance Training

Two studies with resistance training reported reductions in intrahepatic fat. Bacchi et al. [26] demonstrated that moderate-intensity resistance training (three sessions per week, one hour each) led to a substantial decrease in hepatic fat (25.8%, *p* < 0.001) and plasma triglycerides (0.237 mmol/L, *p* < 0.05) after four months intervention. Zelber-Sagi et al. [34] reported that resistance training (three sessions per week, 40 min each) significantly lowered the hepatorenal ultrasound index (HRI) compared to stretching (0.25 vs. −0.05, *p* < 0.05) after 3 months intervention, with a post-intervention HRI score of 1.86 (HRI > 1.5 indicates fatty liver). This group also experienced small but significant reductions in body weight (0.39 kg, *p* < 0.05) and total cholesterol (−0.478 mmol/L, *p* < 0.05). Franco et al. [27] investigated combined resistance and aerobic training, which significantly reduced hepatic steatosis (78.02 CAP, *p* < 0.05). When combined with a low-glycemic index Mediterranean diet (LGIMD), hepatic fat content decreased further (76.37 CAP, *p* < 0.05).

### 2.6. Different Effects of Endurance and Resistance Training

Bacchi and colleagues [26] conducted a head-to-head comparison of resistance and aerobic training in patients with MAFLD over a four-month intervention. Although both modalities reduced hepatic fat, the absence of significant differences limits conclusions about their relative efficacy. Both exercise modalities led to significant absolute and relative reductions in hepatic fat content. However, aerobic training resulted in a greater reduction in intrahepatic fat compared to resistance training (32.8% vs. 25.8%, *p* < 0.001); the between-group difference was not statistically significant [26]. Furthermore, hepatic steatosis is defined as hepatic fat content > 5.56%, resolved in 23.1% of subjects in the aerobic group and 23.5% in the resistance group, highlighting comparable clinical effectiveness.

### 2.7. Effects of Exercise Intensity

Seven studies examined the effects of moderate-intensity training with all but one reporting significant reductions in intrahepatic fat compared to control groups or alternative interventions. Shojaee-Moradie et al. [32], Cuthbertson et al. [30], and Sullivan et al. [33] demonstrated reduced hepatic fat following four-month moderate-intensity aerobic exercise programs when compared to standard care without exercise training. Additionally, Hallsworth et al. [31] found that high-intensity interval training significantly decreased intrahepatic lipid, along with reducing plasma concentration of ALT and AST, in adults with MAFLD, relative to standard care. These improvements occurred without instructed dietary modifications, and participants in the exercise group also achieved weight loss. Abdelbasset et al. [29] compared effects of high-intensity interval training with moderate-intensity continuous training, showing that both interventions caused similar reductions in intrahepatic triglycerides, ALT and HOMA-IR with no significant differences between the two intensities. Overall, although both moderate- and high-intensity exercise showed beneficial effects, differences between intensities were inconsistent and often influenced by concurrent weight loss, limiting firm conclusions about relative efficacy.

Zhang et al. [28] compared a 12-month moderate-intensity exercise program with a 12-month mixed-intensity program that involved 6 months of vigorous exercise followed by 6 months of moderate exercise. Both exercise groups showed reduced intrahepatic triglyceride content (IHTG), as compared to the control group at the 6-month and the 12-month assessments, with the vigorous-moderate exercise group tended to have a larger reduction. However, the difference between the groups appeared to be largely mediated by weight loss. Body weight was significantly reduced in the vigorous-moderate exercise group compared to both moderate exercise and control groups after 6 and 12 months.

Given the heterogeneity of study designs, exercise protocols, and outcome measures, findings across trials should be interpreted with caution, particularly when improvements are observed only within intervention groups and not in between-group comparisons.

## 3. Discussion

The reviewed RCT interventions included adults with MASLD on different training modalities compared to dietary interventions or control groups receiving standard counseling without exercise. All trials reported reduced hepatic fat content after exercise intervention, although one study did not find a statistically significant effect. Reported findings consistently showed improvements in liver fat, insulin resistance, plasma lipids and hepatic enzymes, and body weight. Although the evidence supporting an effect on exercise is well established, this review has several limitations inherent to its narrative design that should be considered when interpreting the findings. The literature search was restricted to a single database (PubMed), which may have led to the omission of relevant studies indexed elsewhere. In addition, no formal risk-of-bias assessment was conducted, and no quantitative synthesis (meta-analysis) was performed.

Our findings support the growing body of evidence that exercise improves metabolic and hepatic outcomes in adults with MASLD; high exercise intensity is associated with greater reductions in IHTG than moderate-intensity training. The EASL-EASD-EASO Clinical Practice Guidelines recommend 3–5 sessions of moderate-intensity aerobic activity per week, totaling 150–200 min, with additional benefits observed from high-intensity physical activity [17]. In line with this, Zhang et al. [28] and Abdelbasset et al. [29] reported that high-intensity exercise promotes the greatest reduction in IHTG levels, and Kistler et al. [35] demonstrated that high intensity is associated with reduced histological severity of MASLD and low prevalence of non-alcoholic steatohepatitis (NASH, now termed MASH).

Despite its benefits, vigorous-intensity exercise may not be sustainable for patients with severe MASLD or low cardiorespiratory fitness. A systematic review reported that simply reducing sedentary time, independent of structured physical activity, was associated with improved MASLD management; Ref. [24] Zhang et al. [28] reported that moderate-intensity exercise provided nearly all the benefits of vigorous exercise. The most substantial reductions in liver fat and body weight were observed during the first six months of exercise, regardless of intensity. After 12 months, reduced abdominal obesity and blood pressure were sustained, although only the vigorous-to-moderate intensity group showed a significant reduction in IHTG at one-year follow-up [36]. Notably, the authors reported no significant differences in IHTG changes between different intensity exercise groups or between the moderate-intensity group and control group. Although only high-intensity exercise reduced IHTG, both intensity groups experienced improved metabolic parameters.

Although aerobic exercise often is chosen as part of lifestyle intervention to reduce weight, resistance training also provides important metabolic benefits beyond weight loss alone. The RESOLVE [37] randomized trial demonstrated that a combination of high-resistance and moderate-endurance training promoted a greater reduction in Fatty Liver Index (FLI) as compared to an exercise program of moderate-resistance and moderate-endurance [37]. Furthermore, observations in the MyoGlu study on 22 physically inactive men (40–75 years of age) with and without dysglycemia/overweight, showed that a combined intervention of endurance and strength training for 12 weeks promoted an extensive reduction in hepatic fat as well as visceral depots [38]. These findings underscore the importance of incorporating resistance training into comprehensive lifestyle strategies for managing MASLD.

In most comparative trials, aerobic training promoted greater improvement than resistance training [26,39,40]. However, it remains uncertain whether these differences arise from type of exercise, intensity variations, or total energy expenditure. Both aerobic and resistance exercise effectively reduce hepatic steatosis and improve insulin resistance when carried out at similar frequencies and durations [23]. However, resistance training may lead to improvements in hepatic steatosis with lower intensity and energy expenditure than endurance training, Ref. [23] suggesting that the two exercise modalities might benefit MASLD via slightly different mechanisms. Across the included RCTs, exercise interventions improving hepatic and metabolic outcomes, generally involved moderate-to-vigorous aerobic activity performed several times per week, amounting to roughly 150–200 min of weekly training. High-intensity protocols tended to produce somewhat larger reductions in liver fat, although moderate-intensity exercise also yielded meaningful benefits. Resistance training, performed two to three times per week, was also effective in several studies and may be a feasible option for individuals with lower fitness levels. Although no formal subgroup analyses were conducted, participants with type 2 diabetes appeared to show greater improvements in insulin sensitivity and hepatic fat in trials including this population. These data suggest that exercise programs might be adapted to individual fitness levels and metabolic status. Indeed, a systematic review found that improving MAFLD requires aerobic exercise at 4.8 metabolic equivalents (METs, moderate-to-vigorous intensity, e.g., brisk walking or slow jogging) with 40 min per session, three times a week [23]. In contrast, only 3.5 METs of resistance exercise for 45 min per session, three times a week, are needed for improving hepatic steatosis [23].

None of the included RCTs assessed hepatic molecular biomarkers such as mitochondrial respiration, β-oxidation pathways, or inflammatory cytokines. Only a few studies reported systemic biochemical markers [25,26,30,31,32,33], such as glucose, insulin, or lipid profiles. Thus, the mechanistic explanations discussed below are based on established physiological and preclinical literature rather than direct molecular measurements from the included studies.

The mechanisms by which aerobic exercise improves hepatic steatosis may primarily involve enhanced ATP use in exercising skeletal muscle, improved insulin resistance and mitochondrial β-oxidation, and decreased lipolysis in fat depots, which subsequently reduces hepatic fat accumulation and inflammation [41,42]. In contrast, the physiological mechanisms underlying the benefits of resistance training are less defined. Current evidence suggests that resistance training not only improves MAFLD by increasing ATP use in skeletal muscle but also improves MAFLD by increasing muscle fiber size and the proportion of fast contracting (type II) fibers, which have a higher capacity for glycolytic metabolism [19]. This shift may lead to improved glucose metabolism and overall metabolic function, including increased insulin sensitivity by activation of GLUT4, AMPK, and caveolins [43,44]. These molecules facilitate glucose uptake and improve insulin signaling in skeletal muscle, improving regulation of blood glucose levels more efficiently [44]. With enhanced oxidative capacity, muscle fibers may become more efficient at oxidizing fatty acids during prolonged activities. This adaptation can contribute to overall fat loss, including the reduction in IHTG. In addition to its metabolic benefits, resistance training may promote a large increase in fat-free mass (FFM) [17].

**Figure 1:**
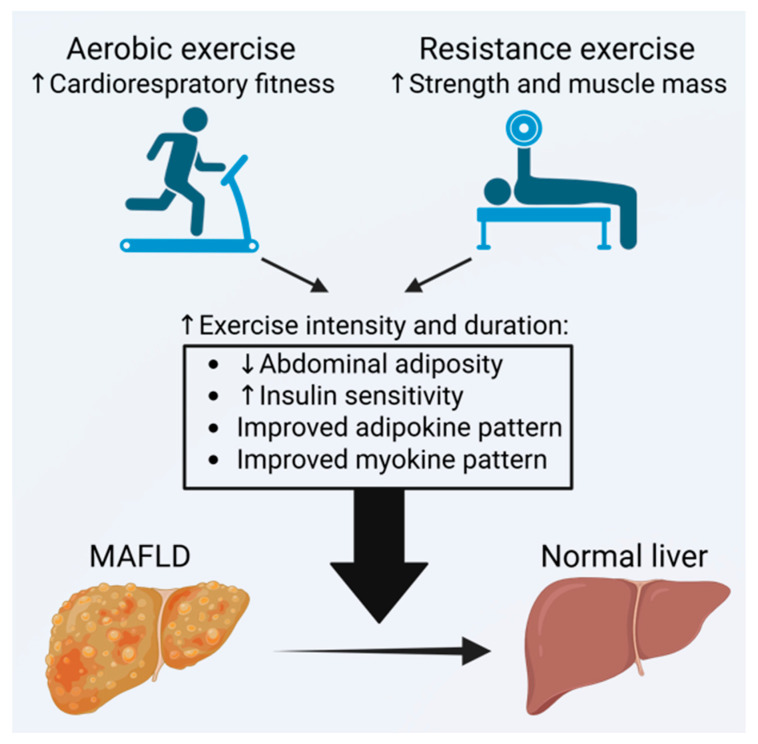
Conceptual framework illustrating the biological mechanisms through which exercise may influence metabolic dysfunction–associated fatty liver disease (MAFLD). Aerobic and resistance exercise improve cardiorespiratory fitness, muscular strength, and muscle mass. Increasing exercise intensity and duration is associated with reductions in abdominal adiposity, enhanced insulin sensitivity, and favorable changes in adipokine and myokine profiles. These combined metabolic and inflammatory adaptations may contribute to decreased hepatic fat accumulation and improvement from MAFLD toward a healthier liver phenotype. The mechanisms depicted are derived from the broader scientific literature and serve as a conceptual overview rather than direct findings from the randomized controlled trials included in this review.

Resistance as well as endurance exercise may improve MAFLD by altering release of signaling molecules from metabolic tissues like skeletal muscle (myokines) and white adipose tissue (adipokines). These signaling molecules may have the potential to improve MAFLD [45], e.g., aerobic exercise can increase serum levels of the adipokine adiponectin, which exerts protective effects against MAFLD [45,46,47]. Aerobic exercise acutely increases secretion of interleukin-6 (IL-6) from skeletal muscle [48]. IL-6 may mediate some of the exercise-induced alleviation of adiposity and hepatic steatosis in mice [49]. Both aerobic and resistance exercise may reduce serum levels of the myokine myostatin [22,50], which may act as an inhibitor of muscle growth and a promoter of liver fibrosis [51,52]. Elevated levels of myostatin are linked to insulin resistance, which subsequently contributes to muscle atrophy. This cycle leads to reduced exercise capacity and various metabolic disorders [51,52]. Collectively, compelling evidence suggests that signaling molecules from skeletal muscle and adipose tissue may play a crucial role in mediating beneficial effects of both aerobic and resistance exercise on MAFLD (Figure 1). These findings suggest that various exercise modalities can exert beneficial effects on hepatic fat accumulation via partially overlapping mechanisms (Figure 1).

For overweight or obese MAFLD patients, a weight loss of 7–10% is recommended to improve hepatic function and related clinical outcomes [17]. However, structured exercise may reduce hepatic steatosis and visceral fat, even in the absence of significant weight loss [26]. Structured resistance and aerobic exercise might even cause a larger loss in IHTG than comparable energy restriction by diet interventions [53]. Although weight loss remains a key target in reducing MAFLD risk among individuals with overweight or obesity, exercise interventions provide a range of health benefits that extend beyond weight reduction alone [21] and should be included as a cornerstone in primary prevention.

In conclusion, although ATP consumed in exercising muscles indirectly benefits intrahepatic triglyceride (IHTG) content, each exercise modality may offer advantages via distinct mechanisms shaped by individual characteristics, physiological responses, and personal preferences. Given the rising prevalence of MASLD, supervised exercise programs should be prioritized as a key component of its management. Current evidence suggests that moderate-intensity aerobic exercise performed for at least 150 min per week, or vigorous-intensity exercise for 75 min weekly, can reduce hepatic fat and improve insulin sensitivity significantly. Incorporating resistance training 2–3 times per week further enhanced metabolic outcomes by increasing muscle mass and improving glucose utilization. Although the optimal exercise ‘dose’ continues to be defined, combining aerobic and resistance modalities appears to provide the most robust benefits. Further research is essential to determine the precise type, intensity, and duration of exercise required for effective MASLD treatment across diverse populations.

## 4. Methods

A narrative literature search was conducted in PubMed from database inception to February 2025 to identify randomized controlled trials evaluating the effects of exercise interventions on metabolic dysfunction-associated steatotic liver disease (MASLD), previously termed non-alcoholic fatty liver disease (NAFLD). The search included combinations of the following terms: metabolic associated fatty liver disease, non-alcoholic fatty liver disease, exercise, physical activity, and training. Additional relevant studies were identified through manual screening of reference lists.

Eligible studies were randomized controlled trials enrolling adults with MASLD/NAFLD and reporting outcomes related to hepatic fat content or metabolic parameters following an exercise-based intervention. Studies were excluded if outcomes were unrelated to MASLD/NAFLD or if combined lifestyle interventions did not allow the independent effect of exercise to be evaluated. Titles and abstracts were first screened for relevance, after which potentially eligible articles underwent full-text assessment to determine alignment with the review focus. Because this is a narrative review, study selection and data extraction were performed with the aim of summarizing key study characteristics and findings rather than producing a systematic or quantitative synthesis.

In total, 11 randomized controlled trials met the eligibility criteria and were included in the qualitative synthesis. Extracted information included participant characteristics, intervention type and duration, comparator conditions, and reported outcomes. The primary outcome of interest was hepatic fat content assessed by imaging or histological methods, with secondary outcomes including liver enzymes, lipid profiles, and indices of insulin resistance or glycemic control. Given the heterogeneity of exercise modalities, intervention durations, and outcome measures, findings were synthesized narratively. A formal risk-of-bias assessment was not undertaken; instead, methodological strengths and limitations of the included studies are discussed qualitatively in the Discussion section.

## Data Availability

No new data were created or analyzed in this study.

## Supplementary Materials

The following supporting information can be downloaded at: https://www.mdpi.com/article/10.3390/ijms27072941/s1.

## Abbreviations

The following abbreviations are used in this manuscript:

ALT	Alanine aminotransferase
AST	Aspartate aminotransferase
BMI	Body mass index
CAP	Controlled attenuation parameter
CH_2_/H_2_O	Proton density fat fraction ratio (MRS metric)
FFAs	Free fatty acids
FLI	Fatty Liver Index
HIIT	High-intensity interval training
HOMA-IR	Homeostatic Model Assessment of Insulin Resistance
HOMA2-IR	Homeostatic Model Assessment 2 of Insulin Resistance
HRI	Hepatorenal index
IHL	Intrahepatic lipid
IHCL	Intrahepatocellular lipids
IHTG	Intrahepatic triglycerides
LDL	Low-density lipoprotein
LGIMD	Low-glycemic index Mediterranean diet
MAFLD	Metabolic dysfunction-associated fatty liver disease
MASLD	Metabolic dysfunction-associated steatotic liver disease
MASH	Metabolic dysfunction-associated steatohepatitis
METs	Metabolic equivalents
MRI	Magnetic resonance imaging
NA	Not assessed
NAFLD	Non-alcoholic fatty liver disease
NASH	Non-alcoholic steatohepatitis
NS	Non-significant
PA1	Aerobic training program (Franco et al.)
PA2	Combined aerobic + resistance training program (Franco et al.)
RCT	Randomized controlled trial
RT	Resistance training
SLD	Steatotic liver disease
T2DM	Type 2 diabetes mellitus
TG	Triglycerides
VAT	Visceral adipose tissue
