# Supplementary material for: Structured Exercise Interventions and Hepatic–Metabolic Outcomes in Adults with MASLD: A Narrative Review of Randomized Controlled Trials
Source: Int J Mol Sci. 2026 Mar 24;27(7):2941. doi: 10.3390/ijms27072941 (PMC13074135; doi:10.3390/ijms27072941)
Supplement: Supplementary file 1 [file ijms-27-02941-s001.zip › ijms-4185977-supplementary.pdf]

Supplementary Table S1. Characteristics of eligible studies

Reference	*Study population and location*	*Sample size*	*Mean (SD) baseline BMI, kg/m^2*	*Duration*	*MAFLD assessment*	*Type of intervention in exercise and control group*	*Nutrition intervention group*	*Exercise*		
								Frequently	*Intensity*	*Duration*
Shojaee-Moradie et al. [32]	Males, MAFLD, England	27	Exercise: 31,6 (0,8) kg/m^2 Control: 31,7 (1,0) kg/m^2	4 months	US, liver biopsy	Moderate-intensity aerobic training and conventional lifestyle advice (control)	No dietary modification	4-5 x week	Moderate (40-60% HRR)	20-60 min
Zelber-Sagi et al. [34]	MAFLD, Israel	82	Exercise: 30.8 (4.5) Control: 31.3 (4.1)	3 months	US	Resistance training and stretching (control)	No dietary modification	3 x week	Comfortable load (10-12 repetitions)	40 min
Abdelbasset et al. [29]	Obesity, DM, MAFLD	47	HII: 36,3 (4,5) MIC: 36,7 (3,4) Control: 35.9 (5.3)	2 months	MRI	High intensity interval (HII), moderate intensity continuous (MIC) and no intervention (control)	NA	3 x week	High (80-85% of VO2max)	3 x 4min
									Moderate (60-70% of max HR)	40-50 min
Cuthbertson et al. [30]	MAFLD	69	Exercise: 30.6 kg/m^2 Control: 29.7 kg/m^2	4 months	H-MRS	Moderate-intensity aerobic training and conventional counselling (control)	No dietary modification	3-5 week	Moderate (30-60% HRR)	30-45 min
Bacchi et al. [26]	Sedentary, T2DM, MAFLD	31	RE: 28.8 (1.1) kg/m^2 AE: 30.5 (1.0) kg/m^2	4 months	MRI	Resistance training (RE) and aerobic training (AE)	Received dietary recommendation for T2DM patients	3 x week	Moderate	60 min

Franco et al. [27]	Moderate or serve MAFLD	144	LGIMD: 32.9 (4.3) PA1: 32.7 (5.4) LGIMD+PA1: 33.1 (4.2) PA2: 30.8 (3.2) LGIMD+PA2: 34.1 (3.8) Control: 34.1 (5.0)	3 months (also assessed at 45 days)	CAP	Low GI Mediterranean diet (LGIMD), aerobic training (PA1), LGIMD+PA1, resistance and aerobic training (PA2), LGIMD+PA2 and control diet (control)	Mediterranean diet focusing on carbs with low GI. No indications on total calories	Weekly	Moderate (60-70% max HR)	150-180 min
Zhang et al. [28]	Sentral obesity, MAFLD, China	220	Vigorous-moderate: 27.9 (2.7) Moderate: 28.1 (3.3) Control: 28.0 (2.7)	12 months (also assessed 6 months)	H-MRS	Vigorous-moderate exercise, moderate exercise and no intervention (control)	Attended health education	5 x week	Vigorous (65-80% max HR)	30 min
								5 x week	Moderate (45-55% max HR)	30 min
Hallsworth et al. [31]	MAFLD	23	HIIT: 31 (4.0) Control: 31 (5.0)	3 months	MRI, H-MRS	High-intensity interval training (HIIT) and standard care (control)	No dietary modification	3 x week	16-17 RPE	5 x 2-3 min 30-40 min
Sullivan et al. [33]	Obesity, MAFLD, USA	18	Exercise: 37.1 (1.1) Control: 40.0 (2.2)	4 months	H-MRS, stable isotope tracer infusions	Moderate-intensity aerobic training and no intervention	NA	5 x week	Moderate (45-55% VO2 max)	30-60 min
Pugh et al. [25]	Obesity, MAFLD + control group (without MAFLD)	34	Exercise: 31 Control: 30	4 months	H-MRS	Aerobic exercise and conventional care (control)	No dietary modification	4-5 x week	Moderate (45-60% HRR)	30-45 min

NA=not assessed, US=ultrasound, MRI=magnetic resonance imaging, H-MRS=hydrogen-magnetic resonance spectroscopy, CAP=controlled attenuation parameter, HR=heart rate, HRR=heart rate reserve (mac HR – resting HR), T2DM=diabetes mellitus type 2.
